# Role of Nanoimprint Lithography for Strongly Miniaturized Optical Spectrometers

**DOI:** 10.3390/nano11010164

**Published:** 2021-01-11

**Authors:** Hartmut Hillmer, Carsten Woidt, André Istock, Aliaksei Kobylinskiy, Duc Toan Nguyen, Naureen Ahmed, Robert Brunner, Thomas Kusserow

**Affiliations:** 1 Institute of Nanostructure Technologies and Analytics (INA) and Center for Interdisciplinary Nanostructure Science and Technology (CINSaT), University of Kassel, 34132 Kassel, Germany; woidt@ina.uni-kassel.de (C.W.); istock@mailbox.org (A.I.); Aliaksei.Kobylinskiy@eah-jena.de (A.K.); toannd@utc.edu.vn (D.T.N.); naureen_ahmed@ina.uni-kassel.de (N.A.); kusserow@ina.uni-kassel.de (T.K.); 2 Applied Optics, Department SciTec, University of Applied Sciences Jena, Carl-Zeiss-Promenade 2, 07745 Jena, Germany; Robert.Brunner@eah-jena.de

**Keywords:** nanoimprint, photonic nanomaterials, optical spectrometers, interferometers, Fabry–Pérot filters, detector arrays, tunable MEMS, linewidth, miniaturization

## Abstract

Optical spectrometers and sensors have gained enormous importance in metrology and information technology, frequently involving the question of size, resolution, sensitivity, spectral range, efficiency, reliability, and cost. Nanomaterials and nanotechnological fabrication technologies have huge potential to enable an optimization between these demands, which in some cases are counteracting each other. This paper focuses on the visible and near infrared spectral range and on five types of optical sensors (optical spectrometers): classical grating-based miniaturized spectrometers, arrayed waveguide grating devices, static Fabry–Pérot (FP) filter arrays on sensor arrays, tunable microelectromechanical systems (MEMS) FP filter arrays, and MEMS tunable photonic crystal filters. The comparison between this selection of concepts concentrates on (i) linewidth and resolution, (ii) required space for a selected spectral range, (iii) efficiency in using available light, and (iv) potential of nanoimprint for cost reduction and yield increase. The main part of this review deals with our own results in the field of static FP filter arrays and MEMS tunable FP filter arrays. In addition, technology for efficiency boosting to get more of the available light is demonstrated.

## 1. Introduction

Smart system integration and smart personal environments are gaining increasing interest. Photonic methodologies enable applications such as smart sensing and high bitrate information technologies with distinct characteristics of small footprint and lightweight via miniaturization, enabling low-cost, high accuracy, and sufficient efficiency. Optical spectroscopy [1,2,3,4,5,6,7,8,9,10] reveals high sensitivity and remarkable selectivity with wide application fields [1], including industrial production control, environmental monitoring, agriculture growth monitoring, medical diagnosis and medical prevention, and communication technologies, to name a few. Some of the optical sensors use nanomaterials and are already integrated into smart personal environments or have high potential to be integrated into those in the future. However, counter-running demands are often involved, for example, concerning size and resolution.

This review starts with an introduction of optical spectrometers and methodologies of spectroscopy for the ultraviolet (UV), visible (VIS), and near infrared (NIR) spectral wavelengths range. Classical spectrometers [4,8,9,10] use prisms or gratings (diffractive elements) to disperse the studied optical signal in an intensity profile as a function of wavelength (spectrum). The higher the order in grating spectrometers, the higher the resolution but the smaller the intensity. Therefore, in many cases the gratings are blazed—meaning that they exhibit a sawtooth structure—to shift the high intensities from zero order into the first or higher diffraction orders [4]. This is a good compromise to benefit from relatively high resolution and relatively high intensity. Microelectromechanical systems (MEMS) can be used to miniaturize grating spectrometers. Electrostatic MEMS actuation has been demonstrated for different material systems and wavelength ranges to rotate the grating [11,12,13,14].

However, optical spectroscopy can also be based on a different methodology: the interferometry [3,4,15,16,17,18,19]. The three main interferometric methodologies are based on the Michelson, Mach–Zehnder, and Fabry–Pérot principles. There are many others, however, and they can be derived from those three. In the Michelson interferometer [3,4], the incident light beam (input beam) is separated by a beam splitter into two parts of identical intensity. The two beams are reflected by two mirrors, reunified again, and reach the photodiode or the screen as the output beam. Precisely moving one of the two mirrors generates a distinct phase shift between the two beams. Provided that a proper calibration has been done, the interferogram (intensity as a function of the mirror displacement) can be converted into the wavelength range, delivering the spectrum of the input beam. However, high mechanical precision is required. Since the required precision is inversely proportional to the light wavelength, it is less challenging in the NIR compared to the VIS range. Spectroscopy using the Michelson interferometer is very powerful for the NIR, also referred to as Fourier spectroscopy [3], since the interferogram is the Fourier transform of the input signal [3].

The Mach–Zehnder interferometer [18,19] also splits the input beam into two parts. The two branches have different optical lengths (defined as the physical length multiplied by the refractive index). After reunification of the two beams in a beam splitter, the output beam includes characteristic phase shifts between the two beams. The Mach–Zehnder interferometer can be integrated using embedded or ridge waveguides. These integrated Mach–Zehnder interferometers are very successful and have been implemented in many different material systems up to now: Si, InP, dielectric materials (inorganic or organic glass), LiNbO_3_, and polymers, to name a few. Implementing an integrated plate capacitor by integrating one plate above and one below the waveguide, the effective refractive index of the waveguide can be adjusted by an electrical field. This field can be controlled and varied by a voltage applied to the capacitor. Therefore, an interferogram can be generated by varying the voltage. Similar to the Michelson interferometer, the spectrum can be extracted by Fourier transform. Another derivative of the Mach–Zehnder interferometer is the arrayed waveguide grating (AWG) [20,21,22,23,24,25,26]. The AWG is a key component in optical dense wavelength division multiplex (DWDM) components used in present optical communication systems. Multiple branches of different distinct lengths are integrated. These components are also known as phased arrays. Pioneering work on AWGs for optical communication in the 1.55 µm communication band was done by Y. Yoshikuni et al., revealing 50 GHz channel spacings [21,22,23]. The AWG has *M* waveguides (also called channel waveguides) of identical effective refractive index but of different physical lengths. Due to the different lengths, distinct phase shifts exist at the end of the waveguides. After passing a free beam section in which only a waveguide exists in the vertical direction, the light constructively interferes at different positions. *M* waveguides are starting there at different positions as output ports. The waveguides are ordered in a sequence of increasing or decreasing wavelengths. Thus, each waveguide carries light of vacuum wavelength *λi* over an interval Δ*λ_i_*, with *i* = 1…*M*. This represents another spectrometer type. 

The third interferometric spectrometer is based on the Fabry–Pérot (FP) principle [4,27,28,29,30,31,32,33,34,35,36,37,38,39,40,41,42,43,44,45,46,47,48,49,50,51,52,53,54,55,56,57]. Although only two parallel transparent dielectric mirrors are involved, the FP interferometer is also highly complex and involves multipath interference. The part between the two mirrors is called cavity and can be any transparent solid or fluid material. Specific wavelengths reveal standing light waves inside the cavity since they fulfil the FP condition: the cavity length is equal to a multiple of half of the vacuum wavelength divided by the refractive index of the cavity material. These specific wavelengths constitute modes and pass the interferometer at almost 100% of their input intensities. The FP interferometer is also called FP filter and the FP modes are also called filter lines. The wavelengths passing the FP filter show constructive multipath interference at the output. All other wavelengths show destructive interference at the output and are reflected by the FP filter. The spectral range between two neighboring modes (filter lines) is called free spectral range, since wavelength ambiguity is avoided. Parallelly displacing one of the mirrors yields a spectral tuning of the wavelength and constitutes a spectrometer. Using MEMS technology, the tuning of one or both mirrors can be performed by MEMS actuation [37,38,39,40,41,42,43,44,45,46,47,48,49,50,51,52,53,54,55,56].

Applying optical structures in the sub-µm or nanometer range offers additional ways to improve or enhance the properties of microspectrometers. As their size is about two orders of magnitude below the typical dimension of MEMS cantilevers or membranes, integration does not affect the general shape or function of these elements. 

A widely used approach is to structure membranes with 1D or 2D photonic crystal (PC) structures to utilize guided mode resonance (GMR) effects [58,59]. The released layer represents in this case a slab waveguide with a light wave incident in normal direction. Without a PC structure, the free space mode will not couple to a guided mode in the slab, but will be reflected and transmitted at each interface, leading to a typical thin-film spectrum. Adding a pattern with period in the range of the targeted wavelength range will enable resonant coupling of a part of the incident wave to a leaky mode in the slab. Due to its leaky nature, the mode will couple back out of the slab to both vertical directions and superimpose with the remaining incident and transmitted free space mode. The result is a resonance line with Fano shape in reflection and transmission [60]. Tailoring the resonant coupling conditions influences the spectral width, the actual line shape, and polarization selectivity. Elements based on GMR are very compact [61] since they translate vertical periodic patterns of a DBR into horizontal pattern on a single membrane or replace full FP filters by a narrowband Fano resonance in a PC slab. However, their fabrication and application are challenging as they possess a high angle selectivity. They not only require very flat membranes or cantilevers, but also restrict the acceptable angular spectrum of the incident light wave. Thus, the usable divergence and spot size, respectively, are limited.

Breaking the 90° mirror symmetry of the PC base elements results in different coupling conditions in x- and y-direction (in-plane coordinates), thus enabling additional polarization selectivity of the filter element. This is achieved in the simplest case by a line grating (1D PC), though more control over the coupling properties is obtained by 2D PC patterns with, e.g., elliptical [62,63,64] or keyhole [65] shaped elements. Another option to introduce polarization selective properties is the use of subwavelength structures. Interaction with incident waves is in this case described by the effective index method. A pattern design breaking the 90° symmetry will lead to different effective indices for transverse electric (TE) and transverse magnetic (TM) polarization, hence leading to structural birefringence [66].

Nano-optical effects may be as well considered to replace the dispersive elements of spectrometers. Surface-plasmon-polaritons show narrow linewidths and interact efficiently in the optical nearfield, but they suffer from the same tradeoff between downscaling and resolution of grating spectrometers since both are based on angular distribution of the spectral range. Additionally, the standard Kretschmann method of excitation makes miniaturization quite difficult due to its bulky prisms. However, there is a possibility to couple a surface plasmon state to metallic gratings on a scanning MEMS cantilever and read out the influence on the current of an integrated photodiode [67]. Another option to improve the resolution of miniaturized spectrometers is given by the super prism effect in PC structures. Dispersion properties of 2D periodic pattern can be designed to be far stronger than those of a single 1D grating if the shape of the photonic bands near the bandgap is tailored accordingly [68].

Following this general overview about spectroscopy, methodology, and instrumentation, the miniaturization potential of spectrometers is focused for grating spectrometers. Optical grating spectrometers for the UV, VIS, and NIR range in length scale between 3 m and several mm. The optical resolution of the grating spectrometer can roughly be calculated by the product of the diffraction order *n* and the number of illuminated grating periods *N*. Miniaturizing the grating spectrometer normally means to reduce *N*. We cannot shrink the grating period since the wavelength range for the application of interest has to be maintained. The only chance is to reduce the size of the grating, i.e., the number of illuminated grating lines *N*. However, reducing *N* has negative impact on the spectral resolution. The left figure in Table 1 shows the grating mini spectrometer C10988MA-01 from Hamamatsu Photonics, Japan. The second column of Table 1 includes data from further miniaturized grating spectrometers C12880MA and the SMD series C14384MA from the same company [69]. To the best of our knowledge, the C14384MA is currently the smallest grating-based optical mini spectrometer available in the market, but the package dimensions are still in the subcentimeter range. The miniaturization has been obtained by reduction of the grating size, i.e., to illuminate only a rather limited number of grating lines. The spectral range is 540–1050 nm; the corresponding linewidths, i.e., full width at half maximum (FWHM) are 17–25 nm and the respective resolutions are 42–56.

As a selection of alternatives to these mini grating spectrometers, we compare in this review paper four strongly miniaturized optical spectrometers (Table 1): AWG devices [20,21,22,23,24,25,26], static FP filter arrays on a charge-coupled device (CCD) or complementary metal oxide semiconductor (CMOS) sensor arrays [27,28,29,30,31,32,33,34,35,36], tunable MEMS FP filter arrays [37,38,39,40,41,42,43,44,45,46,47,48,49,50,51,52,53,54,55,56] on photodetector (PD) arrays, and tunable MEMS photonic crystal (PC) filters [61,70,71,72,73,74]. At first glance, we identify that all these alternatives are smaller in size and reveal higher resolutions, as well as smaller FWHM than the grating spectrometers. Content of Table 1 will be discussed in detail in Section 4, Section 5, Section 6, Section 7, Section 8, Section 9, Section 10, Section 11 and Section 12. Note that there are many more alternatives than those shown in Table 1, such as plasmonic sensors, MEMS grating spectrometers, or Fourier spectrometers, which also have been miniaturized in the past, but this is beyond the scope of our paper. One of the main aspects of our review is to analyze the potential of nanomaterials and nanoimprint for (i) miniaturizing of the optical spectrometers, (ii) improving their performance, and (iii) lowering their production price.

Our paper is organized as follows: Section 2 introduces fundamental principles of FP filters, FP filter-array-based microspectrometers, and our static FP filter-array-based nanospectrometer. Section 3 reviews the digital fabrication methodology for cavity arrays of different heights, which—in combination with multiple lithography steps—can be used for multiple etching or deposition steps. This methodology is demonstrated for the technological fabrication of 3D nanoimprint templates by digital etching. Section 4 reveals the static FP filter array fabrication in the VIS spectral range demonstrating single nanoimprint over three distributed Bragg reflector (DBR) stacks of different heights. Experimental results of static FP filter arrays in the VIS range are presented in Section 5, and our laboratory demonstrator, including a FP filter array on a detector array with telecentric optics, is presented in Section 6. This section focuses on data processing and evaluation. Section 7 covers the partial compensation of rather low efficiency in using available light. A laboratory demonstration of efficiency boosting is presented by spectral preselection. Section 8 presents the fabrication and characterization of static FP filter arrays for the NIR, followed by Section 9 on fabrication and characterization of MEMS tunable FP filters in the NIR range and the methodology to obtain a defined zero residual layer thickness in 3D nanoimprint. Section 10 gives an estimation of potential space requirements after utmost miniaturization, continued by Section 11 on calculation of the potential space required to cover 400 nm in the VIS and 500 nm NIR spectral range for the optical spectrometers shown in Table 1. Potential limits of 2D and 3D nanoimprint lithography are discussed in Section 12. Section 13 addresses the question of nanoimprint by providing essential advantages in term of fabrication time and cost with regard to the five spectrometers compared in Table 1. This paper concludes on the importance of nanomaterials and nanotechnology on the miniaturization of optical spectrometers, performance improvement, functionality, and cost efficiency.

## 2. Static FP Filter Arrays on Photodetector Arrays

### 2.1. Microspectrometers

An FP filter consists of two highly reflective mirrors and a transparent resonance cavity layer in between. The transmitted narrow spectral band is indicated as filter transmission line and the spectral transmission peak is the maximum of this line. Its spectral position is related to the vertical extension or height of the respective cavity—the higher the cavity, the longer the wavelength of the transmitted peak. The highly reflective mirrors are predominantly dielectric DBRs. Each DBR comprises a periodic stack of alternating thin films with low and high optical refractive indices. It can form a high reflective spectral band—the so-called stopband. The stopband width is determined by the contrast of refractive indices of the two chosen materials, i.e., the larger the contrast, the wider the stopband.

A microspectrometer is the combination of FP filter array and corresponding detector array. Since the size reduction of conventional grating-based spectrometers is accompanied by an enormous decrease in optical resolution, the FP filter-array-based spectrometers are an interesting alternative because they do not suffer from resolution decrease [27,28,29,30]. However, these existing FP filter-array-based microspectrometers [27,28,29,30] may require complicated fabrication steps for patterning of various 3D filter cavities with different heights, which is not cost efficient for industrial fabrication. Up to now, different methodologies have been applied to define the different cavity heights, i.e., the resonance cavities of FP filter arrays. Correia et al. [27,28] used *digital etching* to create 16 different FP cavity heights with four lithography/etching steps (16 pixels). Wang et al. 2007 [30] used *digital deposition* to create 128 different FP cavity heights with nine lithography/deposition steps (128 pixels). The *digital masking* shown in Section 3 enables *digital etching* if it is combined with subsequent etching steps [27,28], and enables *digital deposition* if it is combined with subsequent deposition steps [30]. It is obvious, the more different cavity heights that exist in an FP filter array, the more filter transmission lines can be obtained.

### 2.2. Nanospectrometers

**Figure 1:**
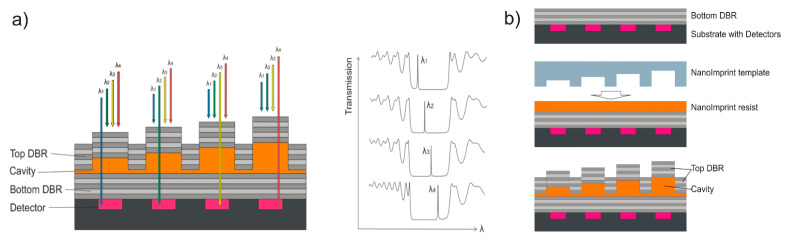
(**a**) Schematic design of a Fabry–Pérot filter-array-based nanospectrometer (left) and its corresponding calculated transmission spectra (right). (**b**) Fabrication process involving substrate conformal imprint lithography (SCIL).

For the purpose of simplifying the fabrication of cavities, we proposed a FP filter array [31,32,33], which contains nanoimprinted transparent resonance cavities with distinct heights and two identical high-optical-quality DBRs. Since nanoimprint technology is used, we introduced it as nanospectrometer, which combines a FP filter array and a detector array. Figure 1a depicts four FP filters as a part of the whole array. A filter array consists of two identical dielectric DBRs (gray) and nanoimprinted transparent cavities (shown orange). Each individual cavity height corresponds to a half wavelength of the filter line in the medium. Thus, each height determines the wavelength of the individual narrow transmission line of the specific single filter. The filter array will be directly deposited on a detector array (black and magenta). Each FP filter corresponds to one detector (Figure 1a). However, a single square filter can also correspond to a multiple of detector pixels (4, 9, 16, …). It is important to note that the cavity height determines the spectral position of the filter transmission line within the stopband. The right part of Figure 1a shows the spectral transmission of 4 FP filters with identical range of minimum transmission (stopband) and varied spectral position of the filter line (due to tailored cavity heights). A 3D nanoimprint allows fabrication of all different cavities in a single step. Note that static FP filter arrays discussed in Section 2, Section 3, Section 4, Section 5, Section 6, Section 7, Section 8 and Section 9 refer to fixed cavity heights, in comparison to MEMS tunable FP filter arrays (in Section 9). 

We used 3D nanoimprint with a single imprint step to structure 192 different cavity heights (192 pixels), which otherwise requires extensive and repetitive fabrication steps. This negates the need for time-consuming digital etching [27,28] or digital deposition [30] in the FP filter array fabrication. Besides, it is more cost efficient since the digital etching is transferred into the fabrication of a 3D nanoimprint template, which can be reused multiple times in the production. For more than 16 pixels, application of digital procedures directly in the array fabrication is cost inefficient and unsuitable for industrial fabrication.

Figure 1b displays the main fabrication steps from top to bottom, starting with the bottom DBR deposition. The liquid cavity material (orange) is deposited by spin-coating. The 3D cavity structure is formed by the transparent 3D stamp (light blue) pressed into the cavity material and hardened under UV radiation. After releasing the stamp, the top DBR is deposited. Nanoimprint is a molding technology for patterning deformable materials, usually polymers. Various nanoimprint technologies [75,76,77,78] have been developed for the purpose of generating 2D structures with high lateral resolution. However, for our nanospectrometer, it is also crucial to accurately control the vertical resolution (3D). To enable mass production, a master template (positive) revealing the checkerboard-type arranged mesa structures (different cavity heights) is replicated into numerous identical stamps (negative). The 3D nanoimprint is performed using one of these stamps, forming the orange 3D cavity structures (shown in Figure 1) simultaneously in a single step. Note that the process described here is for a single stopband. In the upcoming Section 3, a proof-of-principle method to simultaneously print with a single nanoimprint step on three different bottom DBR stacks is reported. This involves three different stopbands and prints over vertical steps occurring at the connection faces of the stacks.

We conclude that unlike grating spectrometers, the nanospectrometer is not affected by the decreasing optical resolution due to miniaturization, and it requires only a single step to define the complex 3D cavity layer, independent of the number of pixels.

## 3. Technological Fabrication of 3D Nanoimprint Templates by Digital Etching

The key issue of 3D nanoimprint lithography is the fabrication of the corresponding 3D templates. It is highly challenging to fabricate 3D templates with diverse mesa height levels and height differences in nm range, and accuracies in sub-nm range. It is comparably easier to fabricate 2D templates with a lateral feature that is small enough to challenge the limitation of current available lithography techniques. One e-beam lithography step and one etching step are capable of fabricating 2D structures with an arbitrary pattern in lateral direction and a desired constant height. Fabricating 3D templates, however, is much more challenging due to the crucial third dimension. It can be fabricated by a series of lithography and etching steps, and the number of fabrication cycles can be dramatically reduced, if the heights are distributed in a digital way. This methodology uses digital masking [33] in the lithography and is combined with subsequent etching steps, as follows.

Assume that *n* different heights are required on a “positive” 3D template (i.e., with protruding 3D mesa structures) and each mesa is larger than the next smaller one. Therefore, all the heights can be viewed as an arithmetic sequence. Suppose that the initial term of the arithmetic sequence is *d* and the common difference of successive elements is *a* (henceforth referred to as step), then the nth term *D_n_* is equal to

*D_n_* = *d* + (*n* − 1)*a* (1)

Equation (1) can be further expressed in a digital way:

*D_n_* = *d* + *s_m_* · 2^*m*−1^· *a* + *s*_*m*−1_ · 2^*m*−2^ · *a* + ··· + *s*_2_ ·2^1^ · *a* + *s*_1_ · 2^0^ · *a* (2)

where *m* is the integer part of (*log*_2_(*n*−1)+1) and s is equal to 1 or 0, with 0 representing unprotected area (“open” state), which leads to etching on the corresponding area and 1 representing protected area (“closed” state) of the lithography, which prevents etching on the corresponding area and results in a height equal to the etching depth. As mentioned before, a limited number of cycles of lithography and etching will be implemented to enable all the different heights *D_n_*. The first cycle will be an initial etch for all the heights where each height position is “open” during lithography. For the remaining rounds, etch will play the role of “digital”; i.e., etch is firstly 2^*m*−1^·*a*, then 2^*m*−2^·*a*, and finally *a*, while lithography will determine 1 or 0 of each height position by taking either “closed” or “open” state (each reticle can be viewed as a 1/0 array). Thus, *n* designed heights can be obtained based on Equation (2) using (*m*+1) times lithography and etching processes. It is very important to note that there is no correlation among the distribution of those mesa heights. In other words, the mesa heights can be arbitrarily distributed in lateral direction according to the requirements of the spectrometer design.

**Figure 2:**
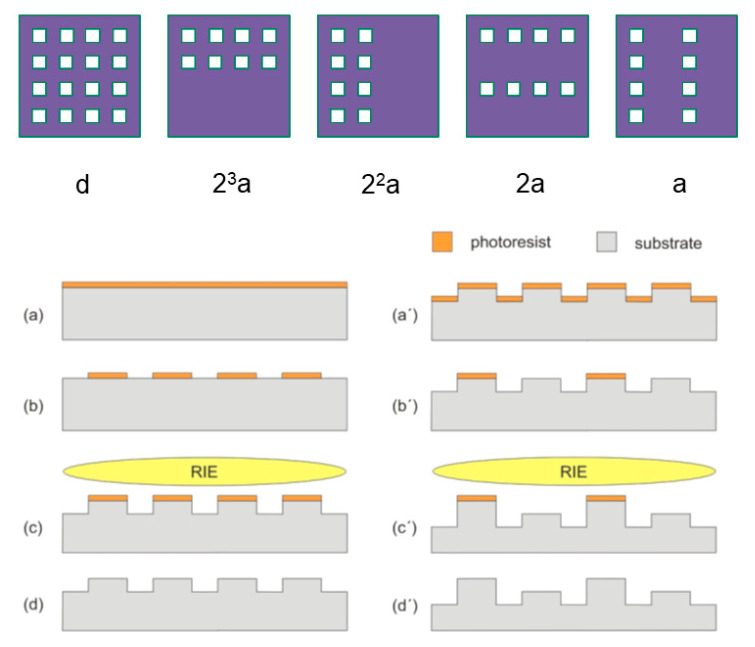
Three-dimensional nanoimprint template fabrication by digital lithography/ etching for a 4 × 4 square array with 16 different heights as an example. (**a**–**d**) The initial height d is created by the first lithography and etching step of all squares. (**a’**–**d’**) Followed by a sequence of four lithography and etchings steps, the heights ranging from the initial height d to (d + 15·a) with the same step a between each other are created. This digital methodology significantly reduces the fabrication steps and saves time and cost, as long as all heights are digital; i.e., they have the identical step or steps with a common divisor. The benefit increases when additional different heights are implemented.

Figure 2 gives a simple example of the digital manner. A 4 × 4 square array with 16 different mesa heights, where the heights range from an initial height *d* to (*d*+15·*a*) with the same step a between each other, can be obtained by five lithography and etching steps in sequence. This digital methodology significantly reduces the fabrication steps and saves time and cost, as long as all heights are digital; i.e., they have the identical step or steps with a common divisor. The benefit increases when additional different heights are implemented; 2*^m^* different mesa heights can be fabricated in (*m*+1) lithography/etching steps (rounds). As proof-of-principle, we fabricated 64 individual mesa heights using seven lithography/etching steps in sequence.

The lateral resolution is mainly defined by the lithography resolution. The vertical resolution is much more critical for fabricating 3D templates using the digital etching methodology. It requires rather high accuracy in etching depth control. Otherwise, the errors of the etching processes will accumulate.

Digital etching is used for defining the different checkerboard-like arranged sinks of different depths (by nanoimprint translated into a checkerboard-like mesa structure of different heights). Correia et al. [27,28] used digital etching for fabricating the checkerboard mesa structure directly and Wang et al. [30] used digital deposition etching for fabricating the checkerboard mesa structure directly. Contrarily, we used it to fabricate the negative of the nanoimprint template, which can be reused multiple times and therefore is suitable for mass production.

## 4. Static FP Filter Array Fabrication in the VIS Spectral Range Demonstrating Single Nanoimprint over Three DBR Stacks of Different Heights

### 4.1. Nanomaterial and Geometric Issues of DBR Mirrors

In the array design prior to the fabrication, the individual cavity heights have been calculated via “OpenFilters” [79] and range from 26 to 215 nm. These cavity heights do not include the residual layer thickness (of about 100 nm). The arrangement of these cavities follows the volume-equalized design [80] to ensure the lateral homogeneity of residual layer as well as possible.

Since DBRs are used as highly reflecting mirrors for FP filters, the transmission lines are only able to be located within the spectral stopband of the DBRs. This means, if a DBR with 100 nm stopband is designed for a FP filter array, all individual filter lines have to be within the stopband of 100 nm, i.e., the detectable range is <100 nm. Since the DBR stopband width depends on the refractive index contrast, we studied different material combinations being deposited via plasma enhanced chemical vapor deposition (PECVD) or ion beam sputtering deposition (IBSD) [33]. SiO_2_/Si_3_N_4_ DBRs result in a spectral stopband width of about 100 nm, while TiO_2_/SiO_2_ DBRs enlarge the spectral stopband to around 200 nm. In summary, the surface quality of resonator cavities, the DBR interfaces, and the constancy of the DBR periods are crucial for the optical quality of the transmission lines (maximum transmissions and minimum linewidths).

### 4.2. Fabrication Process of a FP Filter Array Combining Three Stopbands

**Figure 3:**
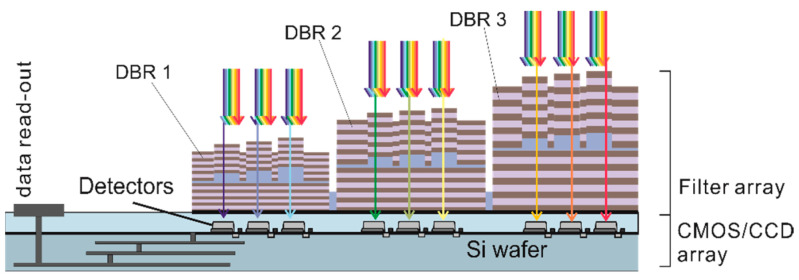
Schematic of a nanospectrometer consisting of an FP filter array with three different distributed Bragg reflectors (DBRs) of different central wavelengths in combination with a sensor array in complementary metal oxide semiconductor (CMOS) or charge-coupled device (CCD) technology. A single step nanoimprint is applied for the definition of the blue cavity layer, providing the term nanospectrometer. In this design type, the FP filter array is placed directly on the photodetector array with processing integrated electronics, below.

In some cases, the spectral width of a single stopband might be too narrow and several stopbands have to be combined to extend the total spectral range of the nanospectrometer. In a proof-of-principle, three spectrally neighboring stopbands were combined, 3D nanoimprint was performed across these three DBR stacks of different heights, and filter arrays were fabricated. For the sake of clarity, Figure 3 depicts a FP filter array consisting of three DBRs of different stopbands with three different FP filters each, which results in a total of nine individual cavities. By means of this method, direct fabrication on a detector chip is possible and no micromounting is required at later stage.

In a DBR, the thicknesses of the thin quarter-wave films define the central wavelengths of the DBR stopbands. For a proof-of-concept, SiO_2_/Si_3_N_4_ DBRs were chosen and deposited by PECVD (requires less than 120 min for a 9.5 period DBR). Structuring of the DBRs is accomplished via lithography and liftoff processes. We demonstrated the methodology with three different stopbands. The more different stopbands are required, the more lithography and liftoff processes are necessary. Therefore, the main challenge for fabricating multiple bottom DBRs is to ensure the quality after several fabrication loops.

Three bottom DBRs of spectrally neighboring stopbands reveal stacks of different vertical heights. The height difference (step height) between DBR 1 and bottom DBR 2 is roughly 224 nm, while the difference between bottom DBR 2 and bottom DBR 3 is 188 nm. The 3D nanoimprint across these vertical steps was done by substrate conformal imprint lithography (SCIL). A hybrid material stamp is used, combining a layer of hard-PDMS (polydimethylsiloxane) for the reason of structure conformity and soft-PDMS as a buffer layer for large-area applicability on a flexible transparent carrier (i.e., thin glass). In general, nanoimprint lithography allows large-area imprints up to 12 inch and SCIL can go up to 8 inch with resolutions down to sub-10 nm [78]. Theoretically, there is no limitation for the number of individual cavities. Note that the SCIL stamp is reusable for up to 500 imprints using hard-PDMS and at least 600 imprints using X-PDMS [78], which definitely eases the fabrication process for individual cavities.

Negative photoresist is used to cover specific parts of the substrate and precisely structure the blank deposition areas before depositing bottom DBR 1. Then the liftoff process is carried out. These processes are repeated for bottom DBR 2 and DBR 3. Next, the 3D cavity layer is defined by a single 3D nanoimprint step, followed by deposition of a specific Si_3_N_4_ protection layer that also acts as the first quarter-wave layer of the top DBRs. The tricky technology involved for this protection layer is reported in [36]. Then, negative photoresist is used again to cover distinct parts of the samples (including three bottom DBRs, the 3D cavity layer, and the protection layer) and precisely structure the blank deposition areas before depositing top DBR 1. Subsequently, liftoff process is carried out. The processes are repeated for top DBR 2 and DBR 3. The negative photoresist AZnLOF diluted by the resist thinner AZEBR (5:1) was used in the photolithography process and NMP as stripping solution was used for the liftoff process.

### 4.3. Lateral Arrangement of the FP Filters within the Array

The composition of one FP filter array is arranged in a 12 × 12 (144) checkerboard-like way. The laterally centered 8 × 8 (64) filters include 64 different cavity heights, while the surrounding 80 filters are used to control the nanoimprint quality and the residual layer thickness. The dimension of each filter (lateral mesa dimensions) is 40 × 40 μm with a distance of 11 μm in between. Therefore, the dimension of each array is about 600 × 600 μm. Such large lateral dimensions have been chosen for the proof-of-principle. In Section 11, the lateral size will be minimized to estimate the potential required minimum space requirements per wavelength span. Since each filter array contains 64 different cavity heights, it reveals 64 distinct transmission lines. Taking into account the three stopbands for each filter array, a total of 192 different transmission lines are obtained. All 192 cavities with different heights are imprinted by one single 3D nanoimprint step, thus, dramatically simplifying the fabrication process.

## 5. Experimental Results of Static FP Filter Arrays in the VIS Range

### 5.1. Transmission Spectra of Static FP Filter Arrays

**Figure 4:**
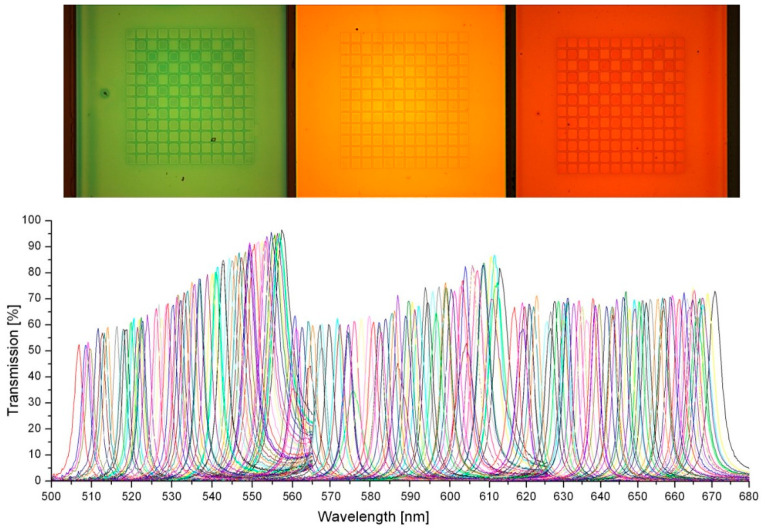
Optical microscope image of three FP filter arrays with corresponding optical transmission spectra below. Reproduced with permission from [36] © Springer Applied Nanoscience, 2018.

The optical spectra of all FP transmission lines were recorded by a microscope spectrometer setup consisting of halogen lamp, confocal microscope, photodetector, lateral active aperture manipulation, and a commercial spectrometer [34]. Figure 4 presents the spectra of 192 transmission lines of the fabricated FP filter array with 3 spectrally neighboring DBRs. The 192 transmission lines continuously cover a spectral range of 163 nm (from 507 to 670 nm) with spectral increments of about 1 nm or below. The highest transmission of all filter lines is measured at 96.5% and the average value of all FP filter line transmissions is 69.6%. The spectral change of the transmission intensities of the subsequent filter lines is due to an interplay of interface roughness, material absorption, reflectivity changes, and so on.

### 5.2. Experimental Linewidths

If we consider only one stopband in the visible range, the transmission linewidth for shorter cavities suffer relatively stronger from the interface roughness than those of higher cavities. The linewidths (FWHM) at shorter wavelengths broaden, thus the transmission intensities are reduced. Additionally, the material absorption at shorter wavelengths is higher than the spectral absorption at longer wavelengths. Therefore, transmission line intensities further decrease with decreasing wavelengths. If we consider multiple stopbands, the thickness of DBR stack layers increases with the increment of central wavelength. Consequently, the DBR with longer central wavelength is thicker than the DBR with shorter central wavelength, which further leads to higher absorption per length.

**Figure 5:**
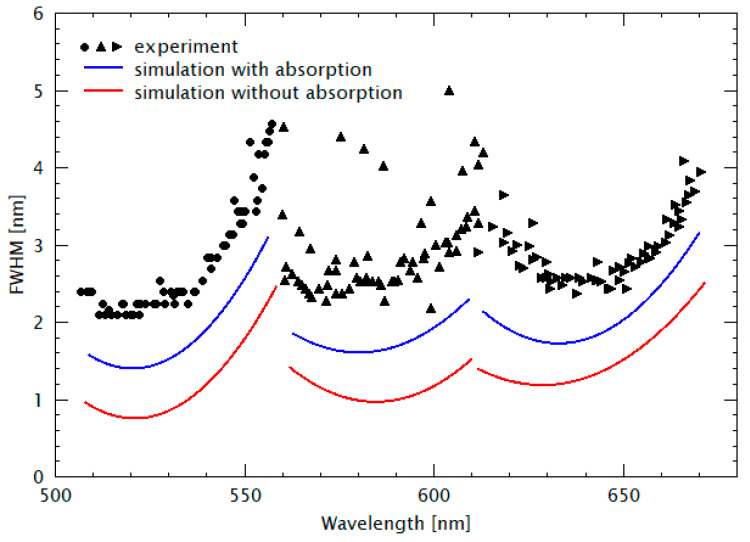
Comparison of measured FWHM (symbols) and simulated FWHM (full lines) of a FP filter array including 3 stopbands for the visible spectral range. The data are taken from the sample shown in Figure 4. The corresponding Transfer Matrix simulations include experimental data for spectral absorption and spectral dispersion. The symbols correspond to experimental data, whereas the full lines correspond to Transfer Matrix model simulations with consideration of the experimental data from spectroscopic ellipsometry. The blue (red) is calculated with (without) spectral absorption. For SiO_2_, the material refractive index *n* ranges from 1.4481 (500 nm) to 1.4358 (670 nm) and the absorption coefficient *α* ranges from 20.3 cm^−1^ (510 nm) to 10.7 cm^−1^ (670 nm). For Si_3_N_4_, the material refractive index *n* ranges from 1.8276 (500 nm) to 1.8019 (670 nm) and the absorption coefficient *α* ranges from 228 cm^−1^ (500 nm) to 118 cm^−1^ (670 nm). For the resist, material dispersion is considered; the material refractive index *n* ranges from 1.53636 (505 nm) to 1.52625 (670 nm).

**Figure 6:**
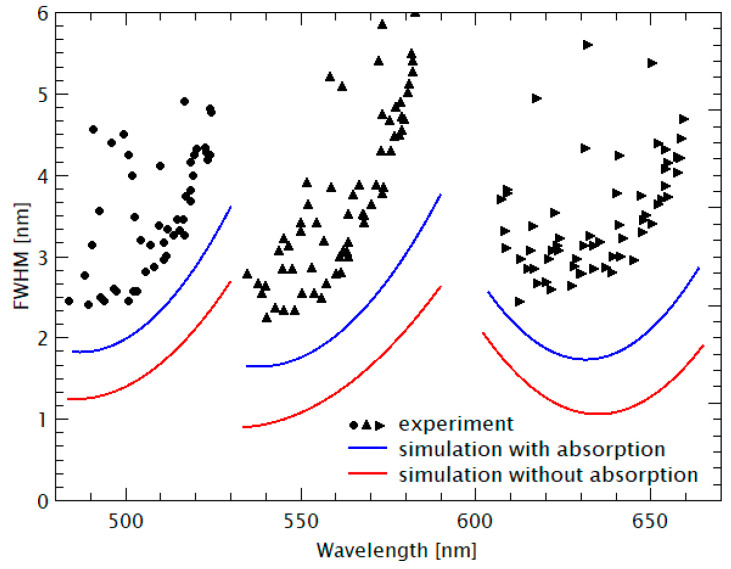
Comparison of measured FWHM (symbols) and simulated FWHM (full lines) of a FP filter array including three stopbands for the visible spectral range which are intentionally shifted compared to Figure 5. The corresponding Transfer Matrix simulations include experimental data for spectral absorption and spectral dispersion. The symbols correspond to experimental data, while the full lines correspond to Transfer Matrix model simulations with consideration of the experimental data from spectroscopic ellipsometry. The blue (red) is calculated with (without) spectral absorption. For SiO_2_, the material refractive index *n* ranges from 1.4505 (480 nm) to 1.4363 (660 nm) and the absorption coefficient *α* ranges from 22.30 cm^−1^ (480 nm) to 11.04 cm^−1^ (660 nm). For Si_3_N_4_, the material refractive index n ranges from 1.8328 (480 nm) to 1.8029 (660 nm) and the absorption coefficient α ranges from 250.39 cm^–1^ (480 nm) to 122.50 cm^−1^ (660 nm). Material dispersion for the resist is considered; the material refractive index n ranges from 1.5391 (480 nm) to 1.5266 (660 nm).

The corresponding FWHMs are displayed in Figure 5. The symbols represent the experimental data and the full lines represent the results of theoretical model calculations using “OpenFilters”, which is based on the Transfer Matrix method considering our spectroscopic ellipsometer measurements of spectral material dispersion *n*(*λ*) and spectral absorption *α*(*λ*). As already mentioned, the wavelength *λ* always denotes the vacuum wavelength. Data from another sample in which the design wavelength had been shifted are depicted in Figure 6.

### 5.3. Discussion of the Linewidth Variation with Spectral Position

Next, the experimental FWHM data are discussed, followed by the estimation of limits for the linewidths at the end of this section. In Figure 5, the experimental FWHM ranges between 1.7 and 5 nm with an average of 3 nm. Larger values are obtained for the filter transmission lines located at the borders of the stopband. The reason for this FWHM variation is due to the fact that the reflectivity of the DBR mirrors is largest in the center of the stopband. The more the filter lines are shifted towards the borders of the stopband, the lower the reflectivity, and thus the larger the FWHMs of the lines. The experimental FWHMs data are generally larger than the simulated results. This phenomenon is partly due to the fact that in our simulation, we neglected (1) the influence of nonhorizontal wave fronts in the cavities during the measurements, (2) the interface roughness existing in the heterostructure might sum up with the number of cavity layers, (3) the absorption of resist material, and (4) Rayleigh scattering.

Finally, the potential minimum FWHM values of FP filter lines are estimated. These depend on the properties and quality of the involved nanomaterials. The FWHM is decreasing with growing DBR reflectivity, i.e., due to the increasing refractive index contrast in the DBR and increasing number of DBR periods (refer to Figure 5 in [52]). Thus, it is determined by the nanomaterial homogeneity, interface quality, and the choice of the nanomaterial combination. In addition, the FWHM decreases with falling material absorption (absorption coefficients are process dependent). In the experiment we obtained smallest FWHM values of 1 nm [32,33], however, as single imprinted FP samples and not in FP arrays, as depicted in Figure 4, Figure 5 and Figure 6. Presumably, even smaller values of down to 0.5 nm should be possible with 15.5 periods of SiO_2_/TiO_2_, an ion beam deposition (IBD) machine, ultrapure material targets (Si, Ti), and long pumping times. However, this was not the target of our proof-of-principle studies.

## 6. Laboratory Demonstrator of a Static FP Filter Array on a Detector Array with Telecentric Optics: Data Processing and Evaluation

**Figure 7:**
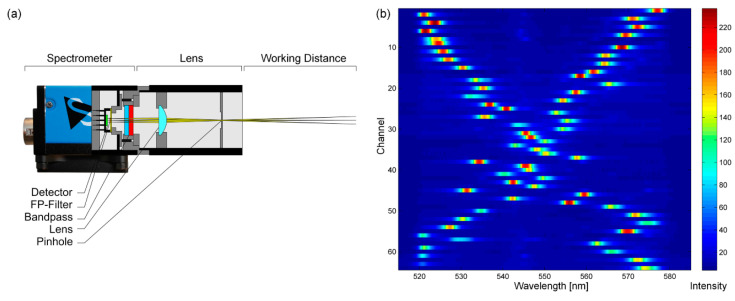
Laboratory demonstrator. (**a**) Schematic cross-section of the demonstrator, consisting of a monochrome CCD camera with integrated FP filter array, bandpass filter and telecentric lens. (**b**) Correlation matrix for 64 detector channels in the spectral interval between 515 to 585 nm. In this design type, the FP filter array is placed on a glass substrate which is aligned bottom-up on a photodiode array. In another design type, the FP filter array is placed directly on the photodetector array (as done in Figure 3).

This proof-of-principle is not yet miniaturized to its potential. A demonstrator was set up by integrating an FP filter array with an optical bandpass into a gray-scale CCD camera (DMK 23U618 by The Imaging Source with Sony ICX618ALA CCD) with telecentric lens (Figure 7a). The signal processing was implemented similar to the work of Emadi et al. [81]. The output signal of the demonstrator is obtained from the image of the CCD sensor. Therefore, the brightness values of the detector pixels below each of the 8 × 8 FP filters (3 × 3 pixels for each filter, with pixel size of 5.6 × 5.6 μm) are recorded as column vector *D* with 64 dimensions (detector signal). The input signal of the demonstrator represents the unknown spectral information *I* of the incident light. The correlation of output and input signal is specified by the correlation matrix *C* as shown in Equations (3) and (4). In the following, *d_M_* represents the signal intensity of the FP filter channel *M* and *i_N_* denotes the intensity of the spectral component *N*.

(3) $$D_{M1}=C_{MN}\cdot I_{N1}\;\left(\begin{array}{c} d_{1}\;\\ \vdots \\ d_{M} \end{array}\right)=\left(\begin{array}{ccc} c_{11} & \cdots & c_{1N}\\ \vdots & \ddots & \vdots \\ c_{M1} & \cdots & c_{MN} \end{array}\right)\cdot \left(\begin{array}{c} i_{1}\\ \vdots \\ i_{N} \end{array}\right)$$

The correlation matrix was experimentally determined by means of a tunable light source (TLS) consisting of a xenon light source and a monochromator (MSH300 by LOT Quantum Design). The demonstrator was illuminated with monochromatic emission of the TLS (FWHM < 1 nm) while the TLS emission was tuned in 0.2 nm steps over the spectral range of the filter element (515–585 nm). The detector signal with 64 channels was recorded for each wavelength step and used to fill the correlation matrix. In the case that the demonstrator is illuminated with the wavelength *i_H_*, the upper equation can be written as

(4) $$\left(\begin{array}{c} d_{1}\\ d_{2}\\ \vdots \\ \vdots \\ \vdots \\ d_{M} \end{array}\right)=\left(\begin{array}{ccccc} c_{11} & \cdots & c_{1H} & \cdots & c_{1N}\\ c_{21} & \cdots & c_{2H} & \cdots & C_{2N}\\ \vdots & \vdots & \vdots & \vdots & \vdots \\ \vdots & \vdots & \vdots & \vdots & \vdots \\ \vdots & \vdots & \vdots & \vdots & \vdots \\ c_{M1} & \cdots & c_{MH} & \cdots & c_{MN} \end{array}\right)\cdot \left(\begin{array}{c} 0\\ 0\\ \vdots \\ i_{H}=1\\ \vdots \\ 0 \end{array}\right)=\left(\begin{array}{c} c_{1H}\\ c_{2H}\\ \vdots \\ \vdots \\ \vdots \\ c_{MH} \end{array}\right)$$

which means that the detector signal for the wavelength *H* is equal to the Hth column of the correlation matrix. Eventually, TLS scan over the full utilizable spectral range of the FP filter array enables us to fill the correlation matrix column by column.

Figure 7b shows the heat map of the recorded correlation matrix for the demonstrator, which was illuminated in the interval from 515 to 585 nm in 0.2 nm steps and recorded with an integration time of the detector of 170 ms. Each of the 64 detector channels on the y-axis shows a sensitivity peak for a certain wavelength depending on the FP filter in front. The mean FWHM for the sensitivity peaks of the correlation matrix is (3.3 ± 0.7) nm.

In the ideal case, given a completely determined square correlation matrix, the intensity distribution of an unknown incident light *I* can be calculated from the detector signal *D*. Therefore, the resulting spectral intensity distribution can be defined as

(5) $$I_{N1}=C_{MN}{}^{-1}\;\cdot D_{M1}$$

from the inverse correlation matrix and the detector signal. Since the correlation matrix is nonsquare and additionally contains minimal irregularities caused by experimental recording, such as detector noise, a least mean square algorithm can be used to determine the spectral information *I*.

## 7. Laboratory Demonstration of Efficiency Boosting by Spectral Preselection

Miniaturized FP filter arrays are superior to miniaturized grating spectrometers with regard to resolution. However, a FP filter array has another disadvantage, namely the light intensity per pixel with wavelength span of Δ*λ* reaching the detector is much less compared to the identical wavelength span Δ*λ* in a grating spectrometer. Following our patent (see Section 14), this disadvantage can be partly compensated by a spectral preselection, which we have recently proven experimentally [82]. This section elaborates on the main outcome of this method.

**Figure 8:**
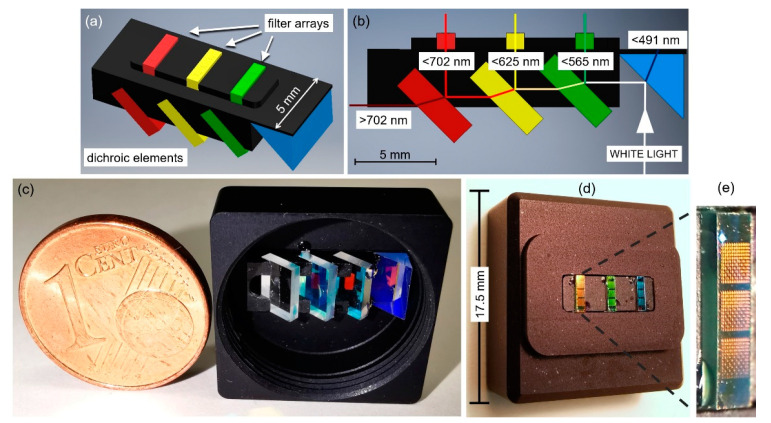
Efficiency enhanced filter-array-based module demonstrating the proof-of-concept of spectral preselection. (**a**) 3D representation of the setup showing the used FP filter arrays and dichroic beam splitters. (**b**) Cross-section of the 3D model indicating transmission and reflection ranges of the beam splitters. (**c**,**d**) Photographs of the module integrated in a mounting for the adaption to a CCD camera. The open front side of the module is shown in (**c**) with a 1 Euro cent coin for size comparison. The backside of the module showing the filter arrays is shown in (**d**), and a magnified image (**e**) of one substrate carrying filter arrays. Reproduced with permission of [82] © The Optical Society, 2020.

FP filter-array-based nanospectrometers can be improved drastically in detection efficiency. In a standard setup of a FP filter-array-based spectrometer, each narrow-band filter of the array receives the entire wavelength range of the light to be analyzed, but only a small fraction of the spectral band passes the filter and can be used in detection. Thus, most of the incident light that reach each filter element is largely reflected. An approach to overcome this drawback is based on the idea to spatially and spectrally redistribute the incoming broadband light across the filter array groups. In this case, each specific filter element receives a preselected spectral partition of the incident light with spatially accumulated intensity. Consequently, reflection and absorption losses are drastically reduced for each filter element. To experimentally verify this concept, an efficiency enhanced spectrometer based on FP filter arrays and dichroic beam splitters has been demonstrated [82]. Figure 8a shows a CAD model of the implemented setup, and its side view in Figure 8b explains the working principle. The incoming light hits a prism, which is the first element of the setup. The prism is a short-pass dichroic beam splitter that reflects wavelengths longer than 491 nm, which include the whole transmission range of the used three FP filter arrays with different DBRs (521–571 nm, 576–630 nm and 628–685 nm). Each of the following three dichroic elements are long-pass beam splitters that reflect the necessary wavelength range to the appropriate filter array and transmits the wavelength range used for the next filter arrays. Shorter and longer wavelengths that are below and above the spectral stopband limit of the filter arrays (shorter than 491 nm and longer than 702 nm) are transmitted accordingly by the first and the fourth element and subsequently absorbed within the housing. The housing is used as a mechanical adapter that connects the optomechanical assembly to a compact CCD camera. The transmitted light passing each FP filter can be read out directly. Figure 8c,d shows photographs of the front and back side of the module (17.5 × 17.5 × 7.8 mm^3^) with all optical elements clearly visible. The module is sealed by a cover with entrance aperture (not shown). A magnified photograph of the glass substrate carrying the filter arrays is presented in Figure 8e. With this approach, an efficiency increase by a factor larger than 3–4 compared to a standard reference system was experimentally demonstrated.

## 8. FP Filter Arrays for the NIR: Fabrication and Characterization

The FP filter arrays are designed for the 1.4–1.5 µm spectral range. For the bottom and top DBR, 9.5 periods of Si_3_N_4_/SiO_2_ are deposited by PECVD. These DBRs are deposited at a temperature of 120 °C to avoid higher temperature exposure to the polymeric 3D cavity layer. The resist mr-NIL210 is used in a single spin-coating process to obtain cavity heights much larger than required in the previous sections for the VIS spectral range. Each filter has lateral dimensions of 40 × 40 µm^2^, and the vertical dimension of the imprinted structures varies between 365 and 530 nm.

**Figure 9:**
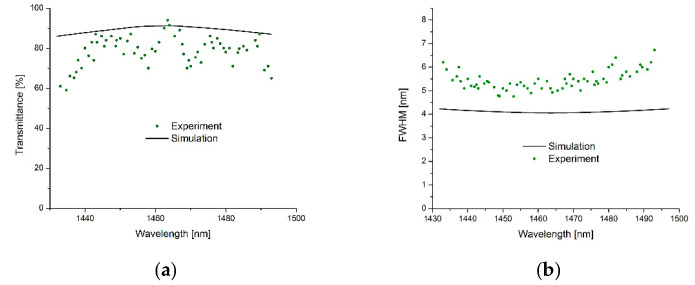
(**a**) Comparison of measured maximum transmission (symbols) of the filter lines of an FP filter array for the NIR spectral range with simulated data (full lines). (**b**) Comparison of measured FWHM (symbols) and simulated FWHM (full lines) of the same FP filter array. The corresponding Transfer Matrix simulations include experimental data for spectral absorption and spectral dispersion. Reproduced with permission from [35] © Springer Applied Nanoscience, 2016.

For optical characterization, a supercontinuum laser, edge filters, two confocal microscope objectives, and a photodiode array are used. Figure 9a depicts optical transmission spectra of FP filter arrays via a selection of some typical filter transmission lines (as representatives) out of 64 spectrally different lines to avoid overloading the figure. High maximum transmission up to 90% and average transmission values well above 70% are observed. The FWHMs of all filter transmission lines are between 4.7 and 8.2 nm and the smallest FWHM is achieved at 1450 nm. This provides a resolution—defined as the ratio of the transmission wavelength and the FWHM—of 300. In Figure 9b, the experimental FWHMs are compared to the results of theoretical model calculations. For the simulation we used “OpenFilters” and experimental material parameters for spectral material dispersion *n*(*λ*) and spectral absorption *α*(*λ*) obtained by spectroscopic ellipsometry. The discussion of the FWHM variation has already been done in Section 5, yet we would like to add some remarks concerning the related interface roughness. The surface roughness of layered heterostructures is investigated on the resist mr-UVCur06 using atomic force microscopy (AFM). We observed an rms value of below 4 nm for a single layer and 8 nm for a double layer. This is qualitatively in agreement with our observations since in layered heterostructures, this measured surface roughness is incorporated and slightly modified into interface roughness. Furthermore, surface roughness and interface roughness between layers exist for each SiO_2_ layer deposited by PECVD. The larger the number of layers included in our cavity, the more interfaces are present, and thus the higher the final accumulation of interface roughness.

Next, the sensitivity of the spectrometer including FP filter arrays against temperature changes is considered. Assuming similar thermal conductivities of the layer materials involved, the interfaces included in the heterostructure might reduce the overall thermal conductivity across the interfaces considerably. A clear and distinct conclusion as well as a full understanding of the impact of interfaces is not yet revealed in the literature [83]. However, most of the studies presented up to now reveal a rule of thumb: the thermal conductivity decreases with rising number of interfaces [83], and the first interface most likely provides the largest heat resistance compared to the last interface of multiple interfaces. Therefore, in the case of only a few interfaces, the difference in thermal resistivity between a single interface and three interfaces is expected to be noticeable. In addition, a multiple cavity layer design may potentially reduce the overall temperature expansion coefficient, if materials of positive and negative coefficients are used in combination. Nonetheless, it is hard to find materials of negative thermal expansion coefficients like yttrium oxide.

## 9. Fabrication and Characterization of MEMS Tunable FP Filters in the NIR Range

**Figure 10:**
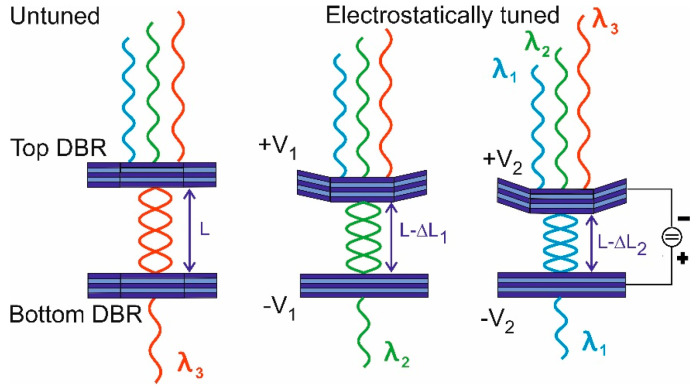
MEMS tunable FP filters with a single airgap. Examples for material systems are GaAs/AlAs, SiO_2_/Si_3_N_4_, or SiO_2_/TiO_2_. In this figure, a four-times half-wavelength (4∙*λ*/2) cavity and electrostatic actuation is shown. The required partial metal layers are not drawn.

Si-based CCD or CMOS photodetector arrays cannot be used for the 1.2–2 µm infrared spectral range, so a more expensive IR detector arrays based on InGaAs must be used instead. To reduce cost of IR spectrometers, MEMS spectrally tunable devices are very attractive. A larger spectral range of 1.15–1.8 µm (650 nm span) can, e.g., be bridged using a small array of only three InGaAs photodiodes and three corresponding MEMS tunable filters, which are spectrally neighbored and each tuning range is over 220 nm respectively. Many different designs of MEMS tunable filters in various material systems have been reported [37,38,39,40,41,42,43,44,45,46,47,48,49,50,51,52,53,54,55,56]. As an example, Figure 12a displays an SEM micrograph top-view of one of our InP-multiple airgap MEMS FP filters; circular central membranes are elastically fixed by four suspensions to four supporting posts. In general, there are many design options for MEMS tunable filters, and two of them are presented in this paper. Figure 10 depicts a first option with a single airgap, where the lower DBR is attached to the substrate (not shown) and remains flat. Only the top DBR is electrostatically or thermally actuated. By varying the airgap cavity height, all incoming wavelengths are reflected except the one constituting a multiple of a half wavelength in the cavity. This wavelength (more precisely a small transmission line with this wavelength in the center) is transmitting the filter. The disadvantages of this design are (i) the relatively thick (and thus stiff) DBR stacks require long suspensions to allow the necessary large membrane displacement and to enable a large tuning range, and (ii) limited stopband widths.

Note that there also other options for MEMS tunable filters based on the thickness actuation of solid components of the filter [57]. However, this review focuses on the thickness actuation of an airgap cavity.

**Figure 11:**
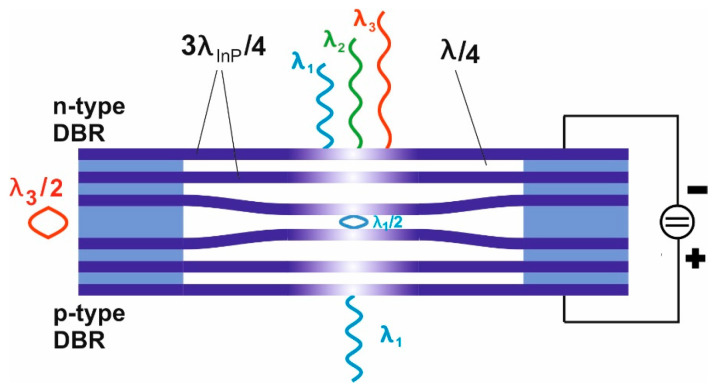
MEMS tunable FP filters with multiple airgaps and InP membranes and suspensions. The supporting posts consist of the corresponding InP/InGaAs multilayer stack. Partly, InGaAs is used as sacrificial layer.

Figure 11 depicts a second airgap-based option that overcomes the abovementioned disadvantage resulting from thick layer stacks (Figure 10). In addition, this second option enables much larger stopband widths. Due to the large refractive index contrast of 1 (air) to 3.167 (InP) between the two DBR materials at *λ* = 1.55 µm, very large stopbands are usable: 500 nm for *λ_air_*/4 with 3*λ_InP_*/4 and even 1500 nm for *λ_air_*/4 with *λ_InP_*/4. A DBR with 2.5 periods of InP/air already provides a reflectivity of 99.8%. The n-doping of top DBR and p-doping of bottom-DBR allows actuation of the airgap cavity via the applied actuation voltage. Our group and others have reported on micromachined InP/airgap DBR-based vertical-cavity tunable filters by different approaches [41,44,45,46,47,48,49,50,51,52,53,54,55,56]. Due to shielding effects, the charges are mainly located at the inside surfaces of the two central membranes. Therefore, only these two membranes are mainly actuated. Comparing this with the “unobtainable situation” where all InP membranes of each DBR are actuated simultaneously, we only suffer from a 10% smaller tuning range, which we derived from detailed theoretical model calculations with a Transfer Matrix-based tool. We fabricated multiple design variants differing in membrane diameter and suspension lengths.

**Figure 12:**
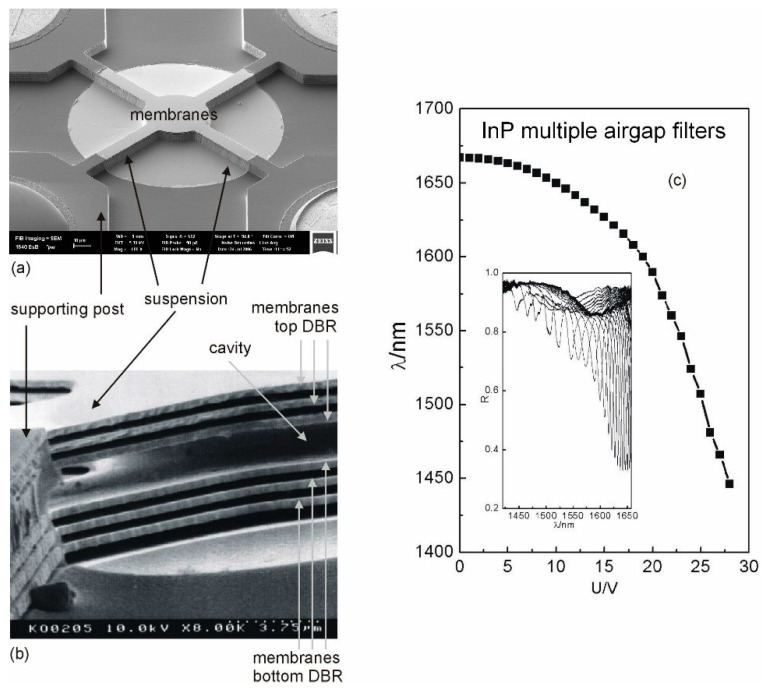
(**a**) SEM micrograph of an InP/InGaAs multiple airgap MEM tunable FP filter. (**b**) SEM micrograph from the side, allowing the view through the airgaps between the suspensions. (**c**) Wavelength of the filter peak wavelengths as a function of actuation voltage. The inset displays a selection of measured reflection spectra *R*(*λ*) recorded as a function of the actuation voltage *U*.

Each DBR consists of 3*λ_InP_*/4 InP membranes and two *λ*/4 airgaps. InGaAs serves as sacrificial layers and parts of the supporting posts. The InP/GaInAs layer stack was epitaxially grown by MOCVD. The filters were fabricated using photolithography and subsequent vertical dry etching (RIE H_2_/CH_4_/Ar), followed by a second lithography to mask the supporting posts. The airgaps and central air were formed by selectively under-etching the GaInAs sacrificial layers using FeCl_3_ etchant. The filter has membranes of 20 µm diameter with four suspensions of 30 µm length and 10 µm width that connect the membrane to supporting posts. SEM micrographs of a filter structure are shown in Figure 12a,b.

The filter (resonant) wavelength as a function of actuation voltage is shown in Figure 12c. The inset shows the corresponding reflectance spectra. The tuning characteristics of the filters show a very large tuning range of 221 nm [55] for the actuation voltage of 28 V. To the best of our knowledge, this is the widest experimental tuning range reported for the InP/multiple airgap DBR-based vertical-cavity filters. The FWHM is narrow (typically 1 nm) at lower actuation voltage; however, it increases at higher actuation voltage due to deformation of the membranes. The record FWHM value that we measured in InP multiple airgap FP filters was 0.1 nm at 1.55 µm. Thus, the potential optical resolution of the FP filter methodology might be 15,000 in the best case.

The InP material system has been chosen to enable monolithic integration with InGaAs photodiodes for telecommunication NIR wavelengths. A laboratory demonstrator with monolithically integrated MEMS tunable FP filters and InGaAs photodiodes was found to be operable. Furthermore, a packaged tunable filter with two fiber pigtails has been demonstrated for the InP/multiple airgap material system.

To avoid the sacrificial layer in 3D nanoimprint, we transferred a technique introduced by Cheng et al. [84] using selective curing in silica templates into our hybrid SCIL templates [85]. Utilization of UV-blocking metal layers enables us to define areas with noncured nanoimprint resist. Thus, cured hard resist remains only in the area of supporting posts, while other areas with noncured resist show a nonexistent residual layer upon the resist removal. This was a breakthrough in nanomaterials and our SCIL 3D nanoimprint lithography [85].

We have shown in our PC MEMS tunable filters the narrowband filters that additionally provide polarization selective properties. This maintains the compactness of MEMS devices by introducing a PC structure with strongly elliptical holes in the top membrane of the top DBR. The most common approaches are to use guided-mode resonance structures [62,63,65] or structural birefringence [66]. The selectivity towards the electric field orientation of the incident wave is in both cases achieved by breaking the 90° symmetry in line gratings or with elliptical elements of photonic crystals.

Zobenica et al. [74] used two evanescently coupled parallel membranes with photonic crystals (PCs) and quantum dots (QDs). Two PC layers replace a single DBR. The early idea is shown in [61,70]. The right-hand graph in Table 1 depicts the MEMS PC membrane design. These devices demonstrated an impressively low FWHM of 0.08 nm at 1319 µm. The spectral filter lines could be tuned over 30 nm by MEMS electrostatic actuation. Positioning and adjusting the QD size for distinct wavelengths is a challenging technology.

## 10. Estimation of Potential Space Requirement after Utmost Miniaturization

Hamamatsu nearly reached the miniaturization limits given by resulting weak resolution values which are still acceptable for some applications. For the proof-of-principle of our VIS and NIR FP filter arrays, we did not miniaturize as much as possible in lateral direction. We chose 40 × 40 µm^2^ mesa sizes in lateral direction for our studies. In the following, estimates are performed concerning the potential miniaturization limits of static and MEMS tunable FP filter arrays, AWG, and MEMS tunable PC filters. Ideal conditions (best case scenarios) for miniaturization and ideal vertical light incidence are assumed. In static arrangements (FP filter array, AWG), we consider Δ*λ* = 1 nm for the channel spacing (spectral step size) between neighboring transmission lines in the VIS range and Δ*λ* = 4 nm channel spacing in the NIR range.

### 10.1. Static FP Filter Arrays to Cover a Spectral Span of 400 nm in the VIS Range

To be on the safe side, the lateral size of the square mesa has to be about 8*λ* × 8*λ* or larger, which we derived by simulations and experiments. Thus, 6 µm side dimensions of the squares are more than enough for an application at *λ* = 600 nm. With a spatial spacing of 4 µm between the mesa, each pixel requires an area of 10 × 10 µm^2^. Using TiO_2_/SiO_2_ DBRs with stopbands of 200 nm, we get 200 spectrally adjacent filter lines per stopband. Therefore, we need two neighboring stopbands to cover the span of 400 nm. With a 100 µm-wide empty frame around each of the two arrays, we require 300 × 350 µm^2^ space, resulting in a chip size of **0.1 mm^2^**. This is well compatible with commercial Si CMOS or CCD detector arrays.

### 10.2. Static FP Filter Arrays to Cover a Spectral Span of 500 nm in the NIR Range

InGaAs photodiodes are much more expensive than Si CMOS or CCD detector arrays. Commercial linear InGaAs photodiode arrays from Hamamatsu have stripelike active areas of 15 × 100 µm^2^ followed by a spacer stripe of 10 × 100 µm^2^. Thus, the period of the stripe/spacer arrangement is 25 µm. This is enough for the abovementioned 8*λ* minimum pixel size at *λ* = 1500 nm. Using TiO_2/_SiO_2_ DBRs with stopbands of 500 nm, we need 125 pixels, which in turn require 100 × 3125 µm^2^. With a 100 µm-wide empty frame around the linear array, we require 300 × 3325 µm^2^ space, revealing a chip size of 1 mm^2^. If noncommercial square photodiodes were used, the required space could be decreased to **0.2 mm^2^**.

### 10.3. MEMS Tunable FP Filter Arrays to Cover a Spectral Span of 500 nm in the NIR Range

InP/multiple airgap DBRs with a stopband width of 500–1500 nm are considered, allowing spectral tuning of >200 nm. Supporting posts of 40 × 40 µm^2^, four suspensions of 40 µm length each, and circular membranes of 15 µm diameter (enough for application at *λ* = 1.55 µm) are arranged. Three neighboring filters are used, and they share the same supporting posts on one side. With an additional 40 µm-wide empty frame around the three filters, we also get 100 µm-wide outer frame as in the last two estimates above, including the inherent nonmembrane areas. This requires 420 × 220 µm^2^ space, revealing a chip of **0.09 mm^2^**.

### 10.4. MEMS Tunable FP Filter Arrays to Cover a Spectral Span of 400 nm in the VIS Range

We consider TiO_2_/SiO_2_ DBRs with a stopband width of 200 nm, allowing 100 nm spectral tuning. Supporting posts of 40 × 40 µm^2^, four suspensions of 40 µm length each and circular membranes of 10 µm diameter (enough for application at *λ* = 600 µm) are arranged. Neighboring filters use the same supporting posts on two sides within the square arrangement of the four filters. With an additional 40 µm-wide empty frame around the three filters, we also get 100 µm-wide outer frame as in the last three estimates above, including the inherent nonmembrane areas. This requires 320 × 320 µm^2^ space, resulting in a chip size of **0.1 mm^2^**. This is due to the dielectric material system exhibiting much smaller tuning ranges with a single airgap MEMS.

### 10.5. MEMS Tunable PC Filter to Cover a Spectral Span of 500 nm in the NIR Range

Fiore et al. [74] reported an experimental tuning range of 30 nm (1308–1338 nm) and mentioned the potential to increase it to 40 nm. Using supporting posts of 15 × 50 µm^2^ and PC membranes with suspensions of 15 × 15 µm^2^, and 15 spectrally neighbored filters with spectral overlap as done above to be on the safe side, we can cover a wavelength span of 500 nm. Similar to the cases above, neighboring MEMS filters use the same supporting posts. Together with the space required for contacts of sensing and actuation diodes, we estimate a chip size of **0.33 mm^2^** is required. We assumed a miniaturization potential of a factor of six, in view of the dimensions of the contacting areas in the Figure 1c in [74].

### 10.6. AWG to Cover a Spectral Span of 500 nm in the NIR Range

Pioneering work on arrayed waveguide gratings (AWGs) has been done by Y. Yoshikuni et al. [23]. They reported on 64 channels of 50 GHz spacing at 1.55 µm requiring only 7 × 3.6 mm^2^ [21,22,23]. They also demonstrated early-on the worldwide most complex InP photonic device integration, including several AWGs, semiconductor optical amplifiers, and photodiode arrays. Based on these early and the most recent publications, we estimated in our comparison, i.e., with 125 channels in the NIR, a space requirement of **50 mm^2^**.

## 11. Resolution Limits of 3D Nanoimprint Lithography

In 2D nanoimprint lithography, the smallest lateral structures of 5 nm have been printed using a MBE-grown GaAs/AlAs heterostructure that is cleaved and selectively etched on the cleaved side, and the side is used as a nanoimprint template [86]. Using a carbon nanotube as a template in the lateral direction, structures as small as 2 nm could be demonstrated [87]. Theoretical model calculations [88,89] were performed, including most of the important forces between all the individual atoms on the nanoscale surface of the template and those atoms on the surface of the imprint resist. This group found that for structures revealing lateral dimensions below 0.5 nm, the template cannot be removed any more from the imprinted and hardened resist. Thus, this could be defined as the limit for size reduction in nanoimprint lithography.

**Figure 13:**
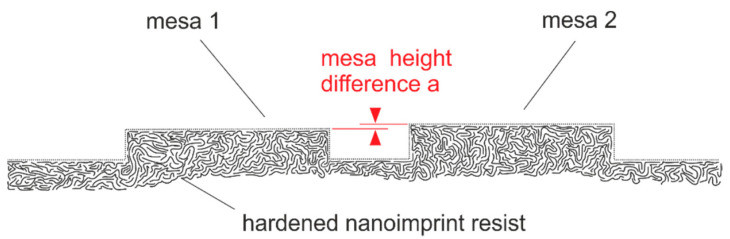
Schematic to visualize the vertical resolution limits in 3D nanoimprint lithography defining mesa structures of different heights. The resist is depicted via schematic organic molecules. The size relations are not to scale: vertical mesa height difference of a = 0.2 nm to 1 nm, lateral mesa width of 6 to 40 µm, and vertical mesa heights 10 to 300 nm. The dotted line is drawn to guide the eye to visualize a 3D surface structure.

However, this result seems to be in contradiction to the smallest height differences we obtained between neighboring mesa structures [32,33] in our templates used for the 3D nanoimprint fabrication of our FP filter arrays. The best transmission lines spectrally are 0.8–1 nm in our experiments. This roughly corresponds to a difference in the spatial cavity layer thickness of 0.5–0.7 nm. Our smallest imprinted spatial vertical height differences that we measured and reproduced are 0.2 nm. This is in no contradiction to the experimental and simulated results above, since the lateral extension of our mesa is in the range of > 6 µm (see Section 10) or even 40 µm in our proof-of-principle (Section 4 and Section 8). The chemical chains of the nanoimprint resist spread laterally. Thus, extremely high vertical accuracy is obtainable at the expense of residual layers and requires complicated reservoir technologies. Even if our zero-residual layer technology [85] is applied, these reservoir technologies and volume equalizing technologies [80] must be applied. Figure 13 depicts a schematic for FP array structures, including two different mesa heights, to visualize the vertical resolution limit in 3D nanoimprint lithography. The polymer chains have different lengths and shape, and they reveal a lateral thickness of about 0.2 nm. The molecules can easily spread and move during the filling of stamp (mold) with resist material. Therefore, we could demonstrate variations in vertical dimensions down to 0.2 nm in our 3D nanoimprints since the lateral dimension were large enough to allow material spreading. As already mentioned above, 2D lateral dimensions down to 2–5 nm experimentally [86,87] and down to 0.5 nm theoretically [88,89] have been demonstrated since the vertical differences in dimension were constant and large enough to allow material spreading. At the moment, we cannot estimate the limits for 3D nanoimprint if ultrasmall variations are involved in lateral and vertical dimension at the same time.

## 12. Role of Nanoimprint for the Five Methodologies Compared

Several molding technologies exist today, such as nanoimprint, injection molding, and LIGA (acronym of the German words **R**öntgen **Li**thographie, **G**alvanik, **A**bformung, which means X-ray lithography, electroplating, molding) [90]. This German acronym is used worldwide. Nanoimprint is molding structures in the range of few nm up to several µm, whereas the LIGA process is molding structures in the range of a few µm to several mm [90]. Injection molding provides larger objects ranging from several µm to the meter range.

Miniaturized grating spectrometers [69] can be produced using injection molding to define grating, curved mirrors, space for the detector array, and housing in a single step. Molding is always a successful strategy to lower production cost. Micrograting spectrometers were also molded using LIGA technology [91]. An integrated InGaInAs heterostructure waveguide in a size of 1 × 1.8 mm^2^ has been fabricated and includes etched transmission grating, etched curved mirrors, and etched guides of optical fibers [92].

This last chapter is to address the question of the role nanoimprint can play in the production of miniaturized spectrometers—specifically AWG, static FP filter arrays, MEMS tunable FP arrays, and MEMS tunable PC arrays, which are compared in Table 1. The advantages of nanoimprint in terms of time, effort, and cost have been advocated and supported from the results presented in previous sections. Except for the classical grating spectrometers, the lithography steps involved in the fabrication of miniaturized spectrometers can be replaced with nanoimprint. However, nanoimprint is not ideal for the accurate positioning of the QDs in the PC structure [74]. This QD positioning is expected to be arduous and will always require, e.g., electron beam lithography. Therefore, nanoimprint lithography might not be first choice fabrication of MEMS tunable PC structures. On the other hand, nanoimprint lithography is highly desirable for AWGs since it has the advantages to replace optical lithography for fabrication in large production volume. Although not absolutely necessary, nanoimprint is also advantageous for fabrication of MEMS tunable FP filter arrays. However, for static FP filter arrays it is essential. Nanoimprint technology is able to mold the whole cavity structure of millions of pixels, and the mold/template can be replicated and reused several times. Theoretically, there is no limit to the number of pixels that can be fabricated in a single cavity layer imprint step [32,33,34,35,36], in contrast to alternative technologies [27,28,29,30]. In a batch process many FP filter arrays can be imprinted at the same time, potentially resulting in substantial cost reduction. The strength of nanoimprint, after all, lies in the potential savings in cost, effort, and time for repetitive process in massive scale, i.e., mass production.

## 13. Conclusions

In our comparison, the MEMS tunable PC filters reveal the lowest filter linewidths of typically 0.1 nm at *λ* = 1.3 µm. The space requirement is also very small. However, combining spectral neighboring tuning ranges to obtain larger spectral spans of 400, for example, might be quite difficult due to the challenging spatial positioning of the QD in the PC crystal, and even more, its challenging spectral adjustment and defined variation from array to array. Conversely, the AWGs also show small linewidths, and the arrangement of several arrays next to each other is feasible. Still, their fabrication for the VIS spectral range is highly challenging.

Typical linewidths of FP filter arrays are larger than the typical values in PC or AWG filters. We only once managed to achieve 0.1 nm linewidth at λ=1.5 µm in InP/multiple airgap FP filters. Acquiring very small linewidths is very challenging in this technology, but on the other hand, it is advantageous in terms of scalability. Many static FP filter arrays can be fabricated next to each other with a single 3D nanoimprint step. This technology promises lowest tentative price per spectral range in the visible spectrum.

Comparing the efficiency to make the most out of available light, the classical grating spectrometer is by far the best in our comparison. However, grating spectrometers suffer much from decreasing spectral resolution with increasing miniaturization. All the other types in our comparison do not suffer from that at all. Their resolution is very high and independent from miniaturization. Secondly, the AWG makes more out of available light than the tunable PC filter array, and the static and tunable FP filter arrays. The latter three have relatively weak efficiencies, but they can be boosted by spectral preselection, as shown in this paper.

Nanoimprint can be applied to all the compared spectrometers, except the classical grating spectrometer. However, the drastic improvement of nanoimprint is only applicable for the FP filter arrays. For a static FP filter array, we demonstrated 192 different filter lines using a single 3D nanoimprint step to structure the complex 3D cavity layer. Although we have successfully demonstrated 192 spectrally different filter lines, theoretically there is no limit to larger values. In addition, nanoimprint can substantially reduce both fabrication cost and effort. The work reported in this paper further concludes that nanomaterials and nanofabrication technologies will play a crucial role for the performance, size, and price of photonic components.

## 14. Patents

For FP filter arrays, the disadvantage of making less use of available light in comparison to grating spectrometers can be partly compensated by spectral preselection: R. Brunner, H. Hillmer and A. Gatto: Spektralsensor zur spektralen Analyse einfallenden Lichts, German Patent 2016, DE 10 2014 108 138 B4. Recently, this has been proven experimentally [54] (Section 7).

## Figures and Tables

**Table 1:**
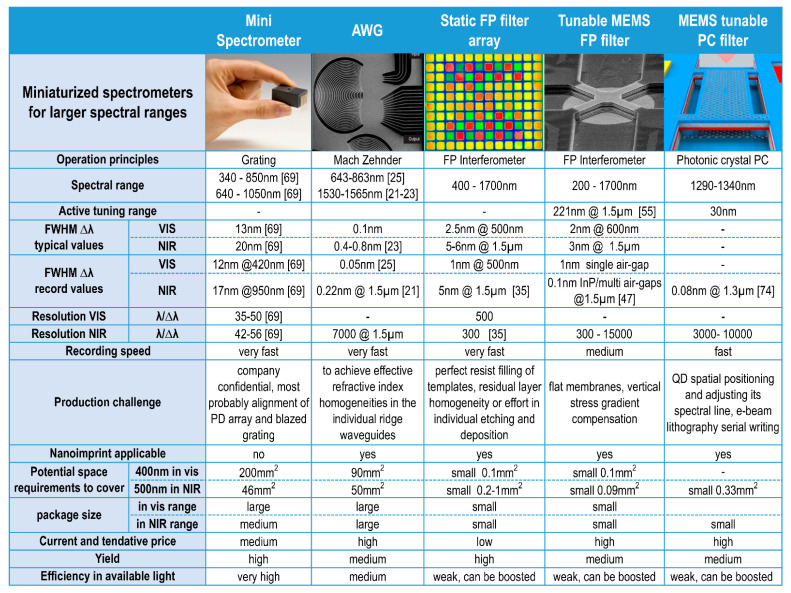
Benchmarking a selection of different miniaturized spectrometers. The characteristics compared comprise mainly optical properties, but also economic and manufacturing points. The wavelength *λ* denotes the vacuum wavelength. AWG = arrayed waveguide grating, FP = Fabry–Pérot, MEMS = microelectromechanical system, QD = quantum dot, FWHM = full width of half maximum.

## Data Availability

We respect and follow the Code of Conduct and the Best Practice Guidelines.
